# Surfactant Proteins SP-A and SP-D Modulate Uterine Contractile Events in ULTR Myometrial Cell Line

**DOI:** 10.1371/journal.pone.0143379

**Published:** 2015-12-07

**Authors:** Georgios Sotiriadis, Eswari Dodagatta-Marri, Lubna Kouser, Fatimah S. Alhamlan, Uday Kishore, Emmanouil Karteris

**Affiliations:** 1 Centre for Infection, Immunity and Disease Mechanisms, College of Health and Life Sciences, Brunel University London, Uxbridge, UB8 3PH, United Kingdom; 2 Department of Infection and Immunity, King Faisal Specialist Hospital and Research Centre, Riyadh, Saudi Arabia; 3 Institute of Environment, Heath and Societies, Brunel University London, Uxbridge, UB8 3PH, United Kingdom; The Hospital for Sick Children and The University of Toronto, CANADA

## Abstract

Pulmonary surfactant proteins SP-A and SP-D are pattern recognition innate immune molecules. However, there is extrapulmonary existence, especially in the amniotic fluid and at the feto-maternal interface. There is sufficient evidence to suggest that SP-A and SP-D are involved in the initiation of labour. This is of great importance given that preterm birth is associated with increased mortality and morbidity. In this study, we investigated the effects of recombinant forms of SP-A and SP-D (rhSP-A and rhSP-D, the comprising of trimeric lectin domain) on contractile events *in vitro*, using a human myometrial cell line (ULTR) as an experimental model. Treatment with rhSP-A or rhSP-D increased the cell velocity, distance travelled and displacement by ULTR cells. rhSP-A and rhSP-D also affected the contractile response of ULTRs when grown on collagen matrices showing reduced surface area. We investigated this effect further by measuring contractility-associated protein (CAP) genes. Treatment with rhSP-A and rhSP-D induced expression of oxytocin receptor (OXTR) and connexin 43 (CX43). In addition, rhSP-A and rhSP-D were able to induce secretion of GROα and IL-8. rhSP-D also induced the expression of IL-6 and IL-6 Ra. We provide evidence that SP-A and SP-D play a key role in modulating events prior to labour by reconditioning the human myometrium and in inducing CAP genes and pro-inflammatory cytokines thus shifting the uterus from a quiescent state to a contractile one.

## Introduction

Preterm labour is one of the leading causes of perinatal mortality and morbidity, and accounts for most new-born deaths as well as in children less than 5 years old [1]. Preterm labour is defined as a birth that takes place prior to the 37^th^ week of gestation. It has been shown to affect approximately 10% of pregnant women, a figure which continues to rise annually, and its causes have not yet been fully elucidated [2]. Premature babies face a high risk of disabilities and impairments, some of which include respiratory illnesses due to improper lung maturation, cerebral palsy, hearing and visual disabilities [3].

Due to the semi-allogenicity of the foetus, it is crucial for the uterine environment to sustain pregnancy. Therefore, there seems to be a cross-talk between pro-inflammatory and anti-inflammatory components for the maintenance of pregnancy and birth [4]. Normal labour is considered to be a pro-inflammatory event, meaning there is a clear shift towards the end of term [5]. Surfactant proteins SP-A and SP-D are collagen-containing, Ca^2+^-dependent C-type lectins, called collectins and are mainly produced by type II alveolar cells and non-ciliated bronchiolar epithelial cells [6]. Their primary structure is characterised by an N-terminal, triple-helical collagen region and a C-terminal carbohydrate recognition domain (CRD) which is trimerised by a coiled-coil neck region between the collagen and the CRD region [7]. This family includes surfactant proteins A (SP-A) and D (SP-D) which can have a dual effect, either anti-inflammatory or pro-inflammatory, depending on their orientation and the receptors they bind to [8].

SP-A and SP-D are part of the innate immune system and play a key role in the maintenance of surfactant homeostasis, pathogen clearance, control of inflammation [9], opsonisation [10], T cell modulation [11], and apoptotic and necrotic cell clearance [12].

There have also been a number of reports confirming an extrapulmonary existence of SP-A and SP-D [7]. SP-A and SP-D have been localised in the human female reproductive tract and the uterus, where they are considered to have a role in protection of the tract from infections [13, 14]. They have been localised in the ovaries, vagina and the cervix [15]. They also appear to regulate foetal lung maturation for respiratory function and may be involved in the maintenance of pregnancy as they have been found to be present in amniotic fluid after 26 weeks of gestation with a clear rise towards term [16]. In foetal lungs surfactant production occurs during the third semester of pregnancy [17]. SP-A and SP-D have been reported in several extrapulmonary tissues and organs such as the digestive system, the brain and the nasal cavity [7].

Condon *et al*., (2004) [18] have shown that a SP-A gestational increase in amniotic fluid was followed by an increase of IL-1β and activation of NF-kβ, thus suggesting a labour initiating role for SP-A in mice. Consistent with the pro-labour properties of SP-A, mice injected with SP-A had preterm delivery, whereas injection of the polyclonal SP-A antibody in the amniotic sac delayed parturition by more than 24 h. SP-D, on the other hand, has been found to interact with decorin, which is the most abundant proteoglycan in foetal membranes and the uterine cervix. This indicates a potential role of SP-D in intrauterine tissue remodelling during parturition [19]. Collectively, there is an emerging consensus on the involvement of SP-A and SP-D during pregnancy and labour. In this study, we sought to investigate the involvement of SP-A and SP-D in the reconditioning, and contractility of the human myometrium, using a human myometrial cell line as an experimental model *in vitro*.

## Materials and Methods

### Expression of the recombinant forms of homotrimeric CRD regions of human SP-A (rhSP-A) and SP-D (rhSP-D)

**Fig 1:**
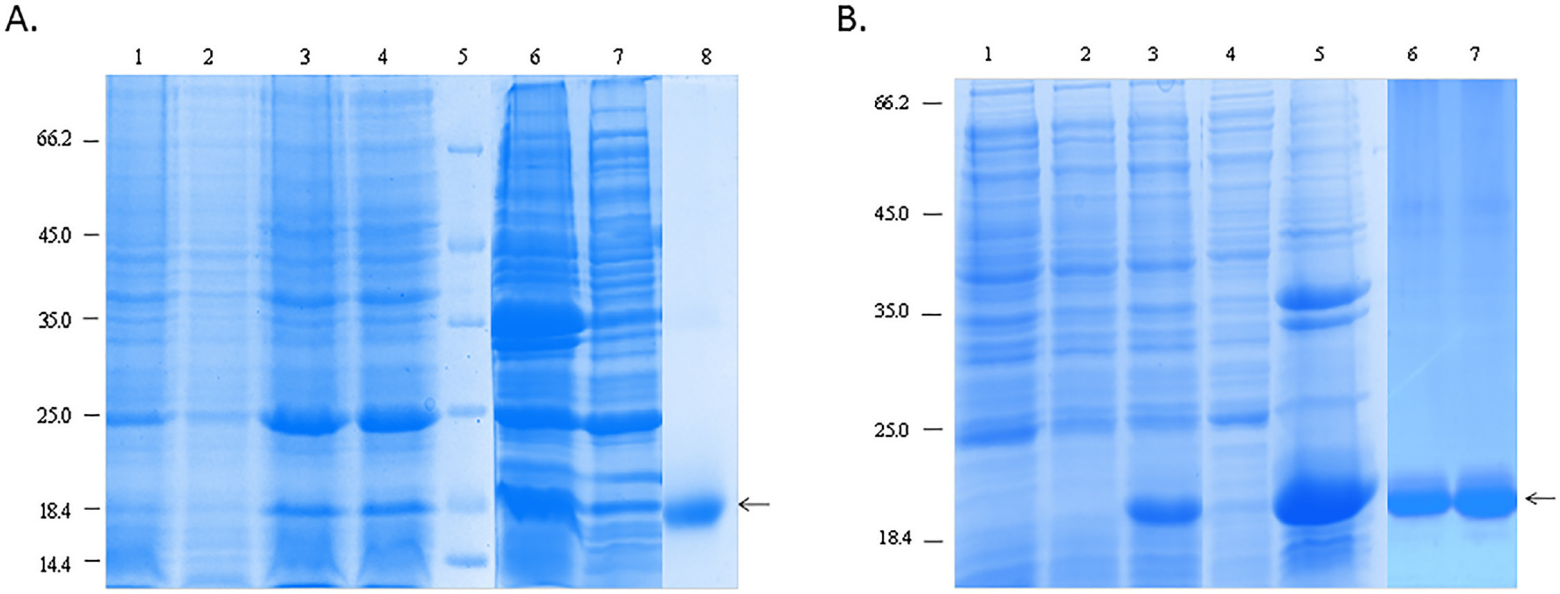
**A.** SDS-PAGE analysis of recombinant SP-A: rhSP-A was expressed in E.coli and purified via maltose agarose column after denaturation and renaturation process. Lane 1 shows, before induction; lane 2, uninduced; lane 3, induced SPA with IPTG; lane 4, induced SPA with IPTG; lane 5, protein marker; lane 6, pellet after lysis; lane 7, supernatant after lysis; lane 8, purified peak fraction of SP-A (arrow) after maltose agarose. **Fig 1B.** SDS-PAGE of recombinant form of SPD: SPD was expressed in *E*.*coli* and purified after denaturation and renaturation process by applying the supernatant to maltose agarose column. Lane 1 shows, before induction; lane 2, uninduced; lane 3, induction with IPTG; lane 4, supernatant after lysis; lane 5, pellet after lysis; lane 6 and 7 peak fractions of rhSP-D (arrow) after maltose-agarose purification.

The recombinant fusion proteins SP-A and SP-D were expressed and purified as described previously [20, 21]. The recombinant proteins containing trimeric lectin domains were expressed in Escherichia coli BL21 (λDE3) pLysS (Life Technologies, UK). The bacterial cells were grown in Luria-Bertani medium with 100 μg/ml ampicillin and 34 μg/ml of chloramphenicol, shaking at 37°C until an A600 of 0.6–0.8 is reached, following an induction with 0.4 mM isopropyl β-D-thiogalactoside (IPTG), left for 3 h shaking at 37°C. The cells were centrifuged 4500rpm, 4°C, for 10 min. The cell pellet was suspended in lysis buffer (50 mM Tris-HCL pH 7.5, 200 mM NaCl, 5 mM EDTA, 0.1% v/v Triton X-100, 0.1 mM PMSF, 50 μg lysozyme) for 1 hour, followed by sonication using a Soniprep 150 (MSE, London, UK) at 60 Hz for 30 seconds with an interval of 2 min (12 cycles), which was then centrifuged at 12000 rpm for 15 min. The pellet was solubilized in 50 ml buffer A (50 mM Tris-HCl pH 7.5, and 100 mM NaCl) with 10 mM 2-mercaptoethanol (Bio-Rad, Hertfordshire, UK) and 8 M urea for 1 hour at 4°C. The soluble fraction was dialysed against a gradient of buffer A containing 4 M urea, 2 M urea, and 1 M urea for 2 h at each urea concentration, which was then dialysed against affinity buffer (50 mM Tris-HCl pH 7.5, 100 mM NaCl, 10 mM CaCl2) externally with 2 changes and then centrifuged (10,000 rpm, 10 min, 4°C). The supernatant was then passed through 5ml column of maltose-agarose column, which was washed 3 column volumes of affinity buffer. Bound SP-D or SP-A were eluted with buffer A consisting of 10 mM EDTA. Samples collected at each stage of purification were analysed on SDS-PAGE (Fig 1), (Figs A-C in S1 File).

5 ml of Polymyxin B agarose gel (Sigma, Poole, UK) in a 20 ml BioRad column was prepared to remove LPS of rhSP-A or rhSP-D. The column was washed with 50 ml of 1% sodium deoxycholate and then washed with 50 ml of sterile dH2O. Peak fractions of rhSP-A or rhSP-D were applied to LPS removal columns for 2 h at 4°C. 1 ml fractions of the flowthrough were collected of the proteins. The levels of endotoxin were determined by QCL-1000 Limulus amebocyte lysate system (BioWhittaker, Walkersville, MD, USA). The concentration of protein fractions was measured at A280 using a NanoDrop. The endotoxin levels were found to be ~ 5 pg μg^-1^ of rhSP-D and ~ 4 pg μg^-1^ of rhSP-A.

Further details of the expression of rhSP-A and its characterisation have been provided as supplementary material.

### Cell culture and treatments

The myometrial cell line ULTR, which was a kind gift from Dr Xiaolan Cui (Department of Obstetrics and Gynecology, University of Cincinnati, USA), and was cultured as previously described [22], in Dulbecco’s modified eagles medium (DMEM) with high glucose (4.5 g/l) and L-glutamine(0.58 g/l) (Gibco, Life Technologies), supplemented with 1% of penicillin and streptomycin along with 10% heat inactivated foetal bovine serum (Gibco, Life Technologies), under standard culture conditions (37° C, 5% CO_2_). Prior to any experiment or treatment, cells were grown to approximately 80–90% confluency. Cells were harvested and a cell count was performed using a haemocytometer. An appropriate amount of cells were seeded depending on the experiment. Cells were treated with rhSP-A and rhSP-D in a dose- (2.5, 5, 10, 20 μg/ml) and time- (4, 6, 12, 24 h) dependant manner.

### RNA extraction and real time PCR

RNA was extracted from treated and untreated ULTR cells using the Nucleospin kit (Macherey-nagel, Bethlehem, USA). RNA was quantified using the Nanodrop software by determining the absorbance at 260 nm. 2 μg of RNA was used to synthesise cDNA using the Precision nanoScript 2 Reverse Transcription kit (Primerdesign, Southampton, UK). A mastermix solution consisting of SYBR green mastermix (Primerdesign, Southampton, UK), sterile water and primers specific for each gene (human SPA-1, SPA-2, SP-D, OXTR, CX43, COX2, mTOR and DEPTOR; Table 1), was added to a 96-well plate and 1 μl of cDNA was added to each well. The plate was then sealed, centrifuged and placed into the ABI FAST HT9000 qPCR machine. Thermocycling conditions for the PCR were denaturation at 98°C for 30 seconds, 30 cycles of [98°C for 10 seconds, 60°C for 30 seconds and 72°C for 30 seconds], and final extension at 72°C for 10 min. Gene expression levels were normalised to GAPDH levels. All samples were analysed in triplicates.

**Table 1:** Primer sequences of human gene targets used for qPCR experiments.

Gene name	Forward primer sequence 5’-3’	Reverse primer sequence 5’-3’	Length in base pairs
SP-A1	TGGGTCGCTGATTTCTTGGA	CGCTGCTCTCACTGACTCA	82
SP-A2	TGAAAAGAAGGAGCAGCGACT	ACCAGGGCTTCCAACACAA	126
SP-D	AATGGCAAGTGGAATGACAGG	CACCCCAGTTGGCTCAGAA	73
OTR	TTACAATCACTAGGATGGCTACAA	CATTTACATTCCCACCAACAATTTAA	105
C43	TGGATTCAGCTTGAGTGCTG	GGTCGCTCTTTCCCTTAACC	130
COX2	CAAATCATCAACACTGCCTCAAT	TCTGGATCTGGAACACTGAATG	89
mTOR	TGCCAACTATCTTCGGAACC	GCTCGCTTCACCTCAAATTC	114
Deptor	CACCATGTGTGTGATGAGCA	TGAAGGTGCGCTCATACTTG	101

### Proliferation assay

Cells were grown as described previously, in 75 cm^2^ flasks and then 150,000 cells were seeded in 6-well plates. Cells were treated with rhSP-A and rhSP-D (10 μg/ml) for 24 and 48 h prior to detachment from the flask and dyed with Trypan Blue. Cells were loaded onto slides and then counted in a Countess Automated Cell Counter (Invitrogen, Life Technologies).

### Immunofluorescence microscopy

70,000 cells were then seeded to a 24-well plate that contained coverslips. Cells were left to adhere for up to 24 h, and then fixed with 4% PFA and permeabilised with 0.5% Triton X-100. Cells were then blocked in 5% FCS/PBS (v/v) and incubated with anti-human SP-A or SP-D polyclonal antibodies diluted in 5% FCS/PBS (v/v) for 45 min. The rabbit anti-human SP-A and SP-d polyclonal antibodies were raised against sequenced verified native SP-A and SP-D (purified from lung lavage) in the laboratory of Prof KBM Reid, MRC Immunochemistry Unit, University of Oxford. Coverslips were washed with 5% FCS/PBS (v/v) before being incubated with a staining solution that consisted of Alexafluor 568 secondary anti-rabbit antibody, phalloidin 488 and Hoechst (Invitrogen, Life Technologies), for 45 min in the dark. After washing with 5% FCS/PBS, the coverslips were mounted on slides and visualised on a HF14 Leica DM4000 microscope.

### ImageStream flow cytometry

A confluent 75cm2 flask of ULTR cells was split evenly into eppendorf tubes, which were centrifuged at 1500 rpm for 5 minutes. The supernatant was discarded and the pellet was washed with sterile PBS, followed by another spin for 3 minutes at 2000 rpm. Cells were fixed in 4% PFA for 7 minutes on ice and then centrifuged for 5 minutes at 1500 rpm. The pellet was washed with PBS and spun at 2000 rpm for 3 minutes. Cells were blocked for 30 minutes in FBS-PBS and then centrifuged at 2000 rpm for 3 minutes. The pellet was incubated overnight with primary antibody (SP-A or SP-D) diluted in FBS-PBS (1:200) at 4°C. After incubation, cells were centrifuged at 2000 rpm for 3 minutes and then washed with PBS. Secondary HRP conjugated anti-rabbit antibody, diluted in FBS-PBS (1:200) was added to the cells for a 30 minute-incubation. The cells were centrifuged and washed once more as described above. Accumax (Sigma) and DRAQ5 were added before visualising in the ImageStream. Compensation samples that only contained antibodies or only DRAQ5, were used for data normalisation. Data and images were analysed using an ImageStreamx Mark II Imaging Flow Cytometer (Amnis, Merck Millipore).

### Live cell imaging

ULTR cells at 80% confluency were detached from the surface of the flask and a specific amount of cells so as to reach confluency between 30–40% was transferred into petri dishes. Cells were treated with rhSP-A or rhSP-D at a final concentration of 10 μg/ml. Cells were left to adhere for approximately 6 h before being placed under a Zeiss Axiovert 200M microscope that had an incubator attached to allow cell survival and to visualise cell motility. After an area with approximately 20–30 cells was found, pictures of the cells were recorded every 5 min for 15 h. The results from the first 8 h were then used to determine cell motility. X and Y coordinates generated through ImageJ were acquired for 25 cells. Distance was calculated using Pythagoras’ theorem, and velocity and displacement were also determined for each treatment.

### Wound healing assay

Cells were grown and detached as mentioned above, transferred to a petri-dish, and then allowed to reach 100% confluency. Using a fine tip, the bottom of each well was scratched in three different places to create a gap. Cells were placed under a Zeiss Axiovert 200M microscope and images were recorded every 5 min over 48 h for untreated as well as treated cells with rhSP-A or rhSP-D.

### Collagen contraction assay

Cells were seeded at a specific density in a 24 well plate. To form the collagen discs as described previously [23], 150,000 cells were mixed with a neutral solution consisting of collagen from bovine skin (Sigma-Aldrich, U.S.A), 10 x PBS and water. The cell-collagen solution was then loaded to a 24-well plate. Wells with no treatments and no cells were used as a control. The plates were incubated for approximately 2 h at 37°C to allow polymerisation. By using a fine pipette tip (1–10μl) and scoring around the edge carefully, the collagen discs were released from the sides of the wells. Cells were treated with rhSP-A or rhSP-D (10 μg/ml). Using a BioRad machine, images were captured post-treatment at zero min (at time of treatment), 3 and 24 h. The software ImageJ was used to measure the surface area changes.

### Myltiplex cytokine array analysis

Concentrations of the following human cytokines and chemokines (CD40, EGF, ENA-78, FGF basic, G-CSF, GM-CSF, GROα, IFN-γ, IL-1α, IL-1β, IL-1ra, IL-1 RII, IL-2, IL-3, IL-4, IL-5, IL-6ra, IL-6, IL-8, IL-10, IL-12 p70, IL-15, IL-17, IL-19, IL-27, IL-31, IP-10, MIP-1α, MIP-1β, TNF-α and VEGF) were measured by MagPix Milliplex kit (EMD Millipore, U.S.A). Magnetic beads coupled to specific analytes were incubated with the supernatant of myometrium cells treated with rhSP-A or rhSP-D at different time points and doses, and assay buffer. The samples were loaded on a 96 well plate and kept at 4°C for 18 h. After a series of washes, detection antibodies were incubated with the magnetic beads for 1 hour at room temperature and then with a Streptavidin-Phycoerythrin conjugate for 30 min. Finally, sheath fluid was added to each well and the plate was using the Luminex Magpix instrument, according to manufacturer’s instructions.

### Statistical analysis

**Fig 2:**
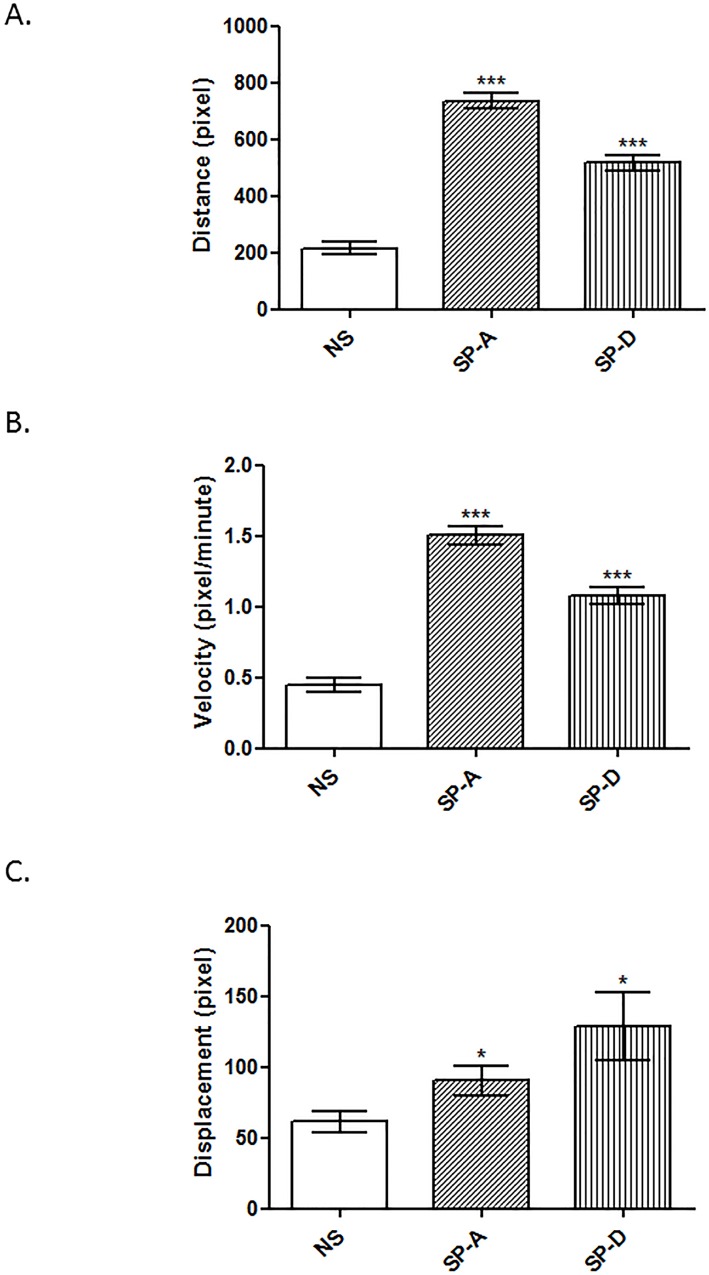
Representation of the average distance (A), velocity (B) and displacement (C) comparisons in ULTR treated with and without rhSP-A and rhSP-D (*p<0.05, **p<0.01, ***p<0.001). Cell movements (n = 25 cells, from a single experiment) were observed either without treatment or with treatment with surfactant proteins (10 μg/ml). Both treatments led to a significant increase in velocity, distance travelled and their displacement from their initial position compared to untreated cells. rhSP-A treatment led to a significant increase in velocity and distance compared to rhSP-D treated cells. There was no apparent significance in displacement in between treatments.

Graphs were compiled and statistical analysis was acquired using Graphpad Prism 5.0. Normal distribution was identified using the f test. If data was equally distributed then to identify significance between treatments and time points for the experiments, a student’s t-test was performed (*p<0.05, **p<0.01, ***p<0.001) between treated and control samples but also between different treatments. When the data was not equally distributed, Mann-Whitney U test was used. Error bars represent the standard deviation for each group of data, apart from Fig 2 where error bars represent the standard error of the mean.

## Results

### SP-A and SP-D affect the cell motility of myometrial cells

During gestation the myometrium goes through a phase of increased production of extracellular matrix proteins and subsequent increase of smooth muscle cell size [24]. It was therefore important to assess the effects of SP-A and SP-D on cell motility. ULTRs were treated with rhSP-A or rhSP-D (10 μg/ml) in a petri-dish and photographs of the cells were taken every 5 min for 12 h. Images from the first 8 h were used for the analysis. Individual cell movements were tracked using ImageJ that provided the coordinates for each point. About 25 cells were tracked for each experiment, including no supplement. Both rhSP-A and rhSP-D were able to increase the distance and the velocity of ULTR cells compared to untreated cells (Fig 2A and 2B); with rhSP-A having a more profound effect on velocity and distance (1.4 fold) than rhSP-D. rhSP-A increase was also significant compared to the rhSP-D treated cells. Moreover, both proteins induced significantly cell displacement (Fig 2C). There was no apparent significance in between treatments.

The human myometrium also differentiates in terms of cell number and size in two distinct stages of pregnancy: hyperplasia and hypertrophy [24]. To investigate the effects of SP-A and SP-D on cell proliferation, ULTRs were treated for 24 h with 10 μg/ml of rhSP-A and rhSP-D. Cell count analyses demonstrated a modest increase of cell proliferation in the rhSP-D treated cells, whereas treatment with rhSP-A did not have any effect compared to control (data not shown).

### SP-A and SP-D augment uterine wound healing

**Fig 3:**
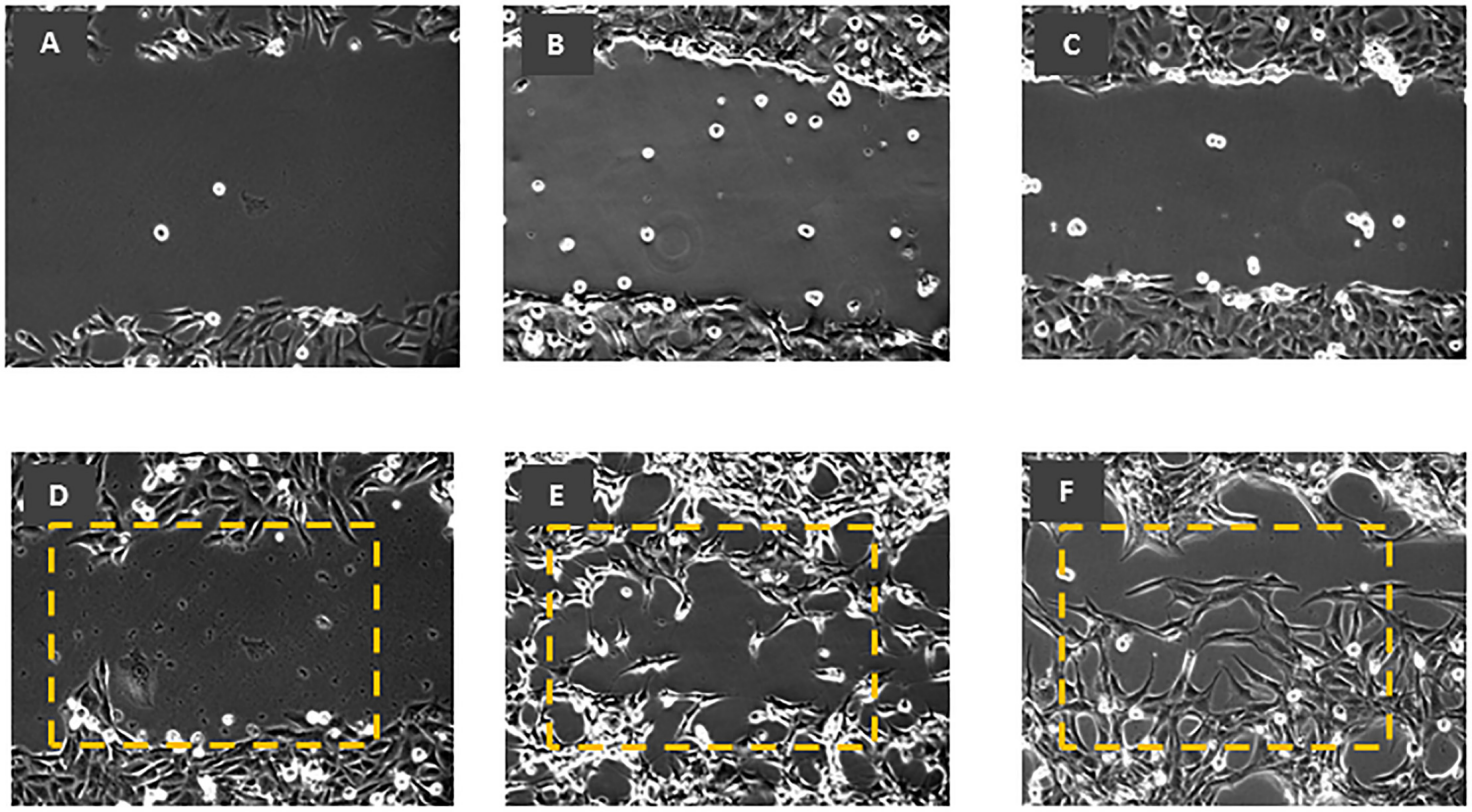
Wound healing assay using myometrium cells untreated (A), and treated with rhSP-A (B) and rhSP-D (C) at 0 and 48 h time points (D, E, and F respectively). Untreated cells (A) failed to close the gap even after 48 h (D), whereas the cells treated with rhSP-A (B) closed the gap after 17 h (E) while those treated with rhSP-D (C) after 25 h (F). Dotted rectangular annotates the area covered by treated ULTRs. Gap that was less than 3 cells apart was considered as closed. x 10 magnification.

Few histopathologic studies on uterine wound healing have been reported. To further investigate the functional effects of surfactant proteins on myometrium cells, a wound was created within the confluent cells growing on a petri-dish. Images of the cells were taken every 10 min for up to 48 h and the distance of the gap was measured. The untreated cells (Fig 3A) failed to close the gap even after 48 h (Fig 3D), whereas the cells treated with rhSP-A (Fig 3B) closed the gap after only 17 h (Fig 3E) and cells treated with rhSP-D (Fig 3C) after 25 h (Fig 3F).

### SP-A and SP-D induce myometrial contractility *in vitro*

**Fig 4:**
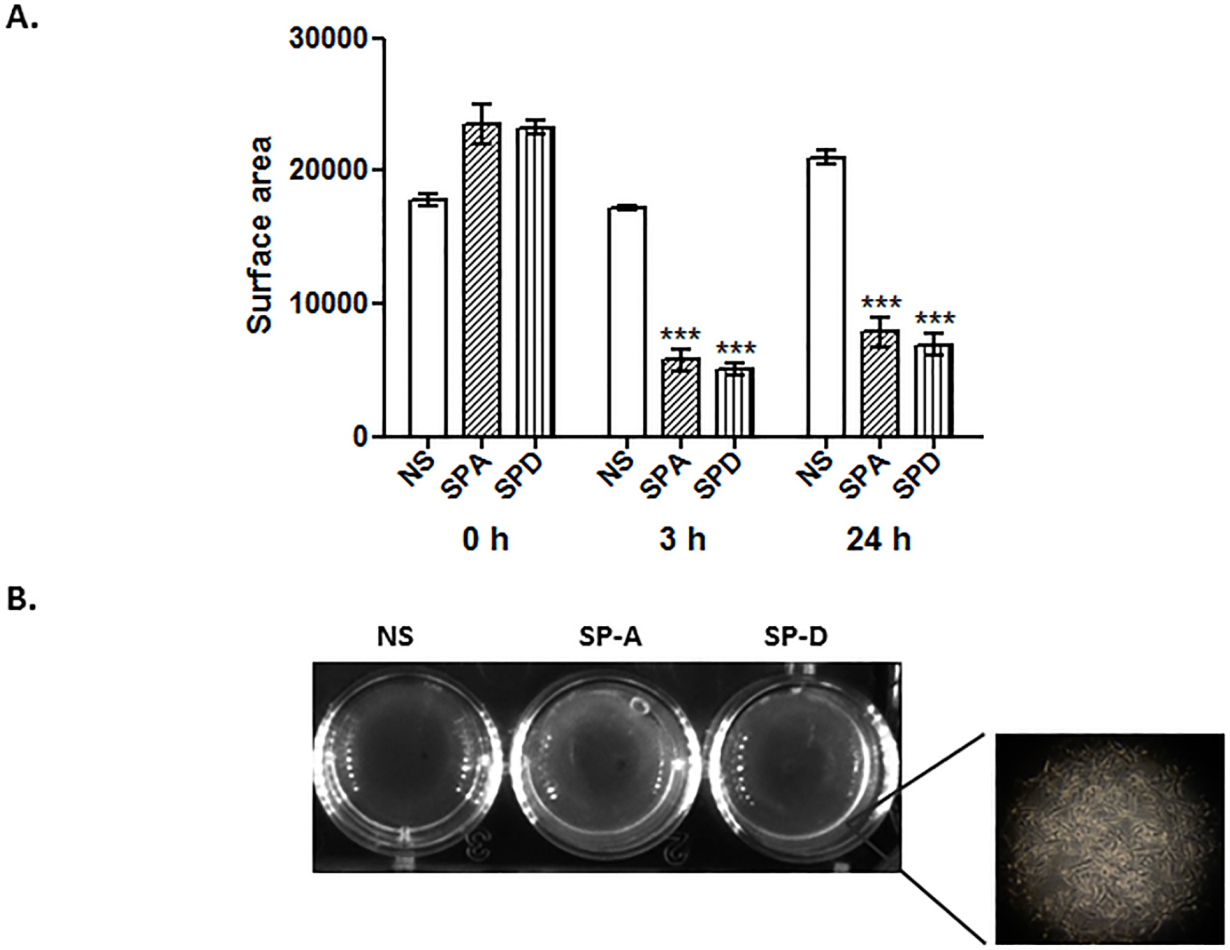
Surface disc area comparisons between myometrium cells growing in collagen with or without treatment with rhSP-A or rhSP-D, after 3 and 24 h. Cells were seeded at a specific density and were grown in a collagen matrix to mimic a 3D milieu. rhSP-A and rhSP-D significantly induced a similar contractility of ULTR cells compared to untreated cells, at 3 and 24 h (A), (*p<0.05, **p<0.01, ***p<0.001). There was no significance in between treatments or time points. Representative collagen discs of 3 h treatment (B; NS: no supplement). Insert: image of ULTRs grown within the collagen matrix (n = 4).

The human myometrium is mostly in a quiescent state during the gestational period and becomes contractile during labour stages. There have been numerous studies regarding the mechanisms involved the transition from quiescent to a contractile state using *in vitro* and *ex vivo* models [25, 2]. To date, no data is available regarding the impact of SP-A and SP-D on this physiological response. Therefore, to assess the effects of rhSP-A and rhSP-D on contractility, cells were grown on 3D collagen matrices, and then treated with 10 μg/ml of rhSP-A or rhSP-D. The surface area of the collagen discs was measured using ImageJ at 3 and 24 h post-treatment. After 3 h, the area of the collagen disc of the cells treated with rhSP-A was 66% smaller, whereas the area of the disc treated with rhSP-D was 70% smaller compared to the untreated cells (Fig 4A). The effect was evident even after 24 h with surface areas similar but only slightly increased (62% and 67% smaller compared to the untreated cells respectively). There was no significant surface area change between treatments or time-points (3 and 24 h) post-treatment.

### Modulation of mRNA expression of pro-labour mediators and genes involved in myometrial reconditioning by SP-A and SP-D

To investigate the effects of surfactant proteins on the contractile machinery we treated ULTR cells with rhSP-A and rhSP-D at different concentrations and RNA was then extracted at 0, 4, 6 and 12 h. We then set out to determine the relative amounts of oxytocin receptor (OXTR), gap junction protein connexin 43 (CX43), cyclo-oxygenase 2 (COX2), mechanistic Target of Rapamycin (mTOR), DEPTOR and human SP-A, SP-D mRNAs. We chose the above mentioned panel of genes since CX43, OXTR and COX2 are pro-labour mediators expressed in human myometrium, whereas mTOR plays an important role in myometrial reconditioning [24]. Finally, we also investigated the impact of these treatments on the expression of SP-A and SP-D themselves.

**Fig 5:**
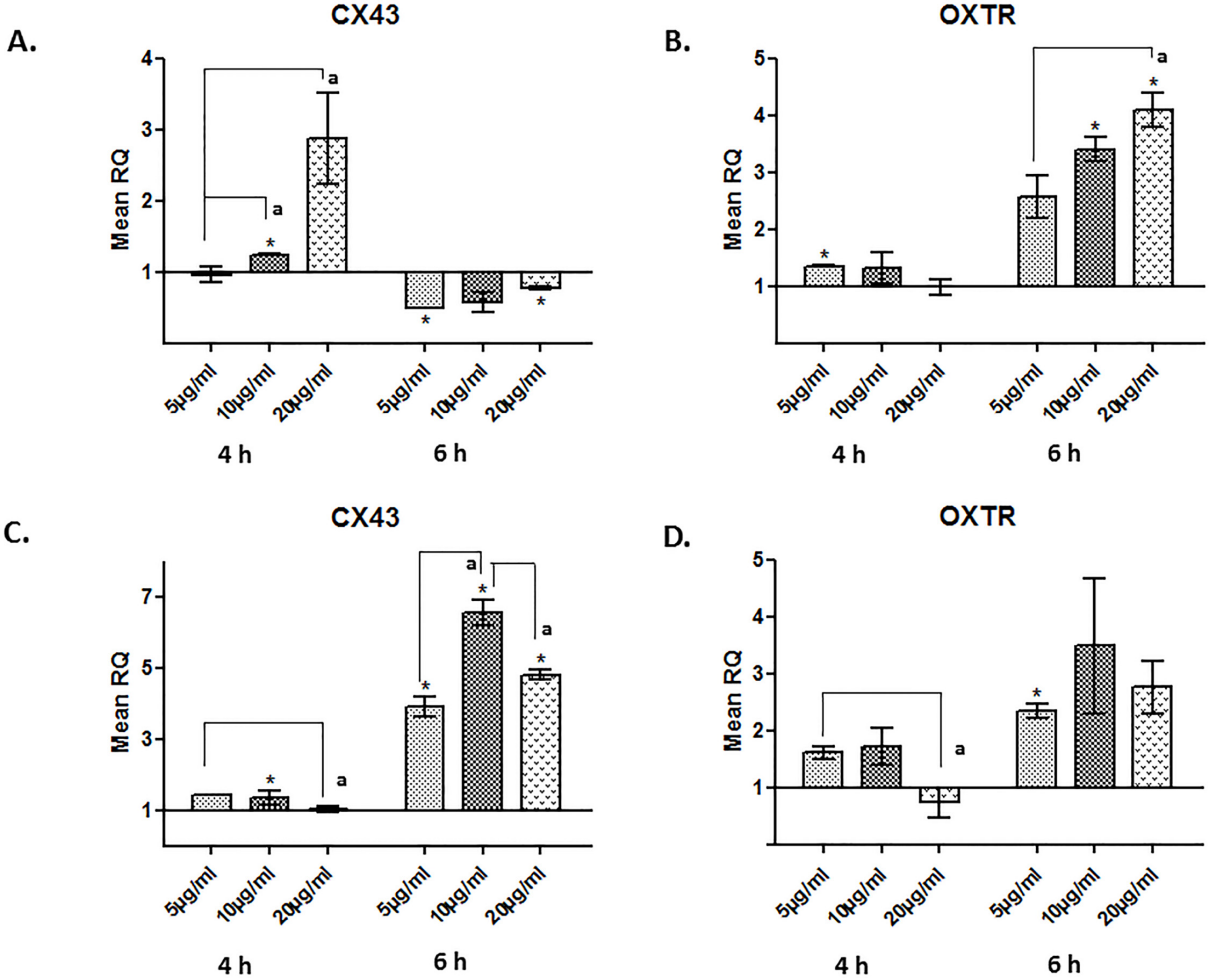
Relative quantification comparisons of CX43 and OXTR in ULTR cells treated with 5, 10 and 20 μg/ml of rhSP-A (A-B) and rhSP-D (C-D) after 4 and 6 h (*p<0.05, **p<0.01, ***p<0.001). rhSP-A led to an increase of CX43 mRNA expression after 4 h at a concentration of 10 and 20 μg/ml, effect that seemed to disappear after 6 h, and of OXTR after 6 h at concentrations of 10 and 20 μg/ml. rhSP-D resulted in an increase of CX43 transcript at all concentrations, and of OXTR transcript after 6 h at a concentration of 5 μg/ml (n = 3). Letter denotes values significantly different from each other as indicated by the horizontal and vertical lines (p<0.05).

rhSP-A treatments resulted in an increase of CX43 mRNA expression at a concentration of 10 and 20 μg/ml after 4 h, compared to the untreated cells, an effect that appeared to disappear after 6 h (Fig 5A). rhSP-A treatment also resulted in an increase of OXTR mRNA at a concentration of 10 and 20 μg/ml after 6 h compared to the untreated cells (Fig 5B). rhSP-D had a more profound effect on the CX43 transcript production at all doses after 6 h of treatment (Fig 5C). rhSP-D treatments led to an increase of OXTR mRNA expression at a concentration of 10 μg/ml after 6 h (Fig 5D), compared to the untreated cells, an effect that appeared to disappear after 12 h (data not shown).

**Fig 6:**
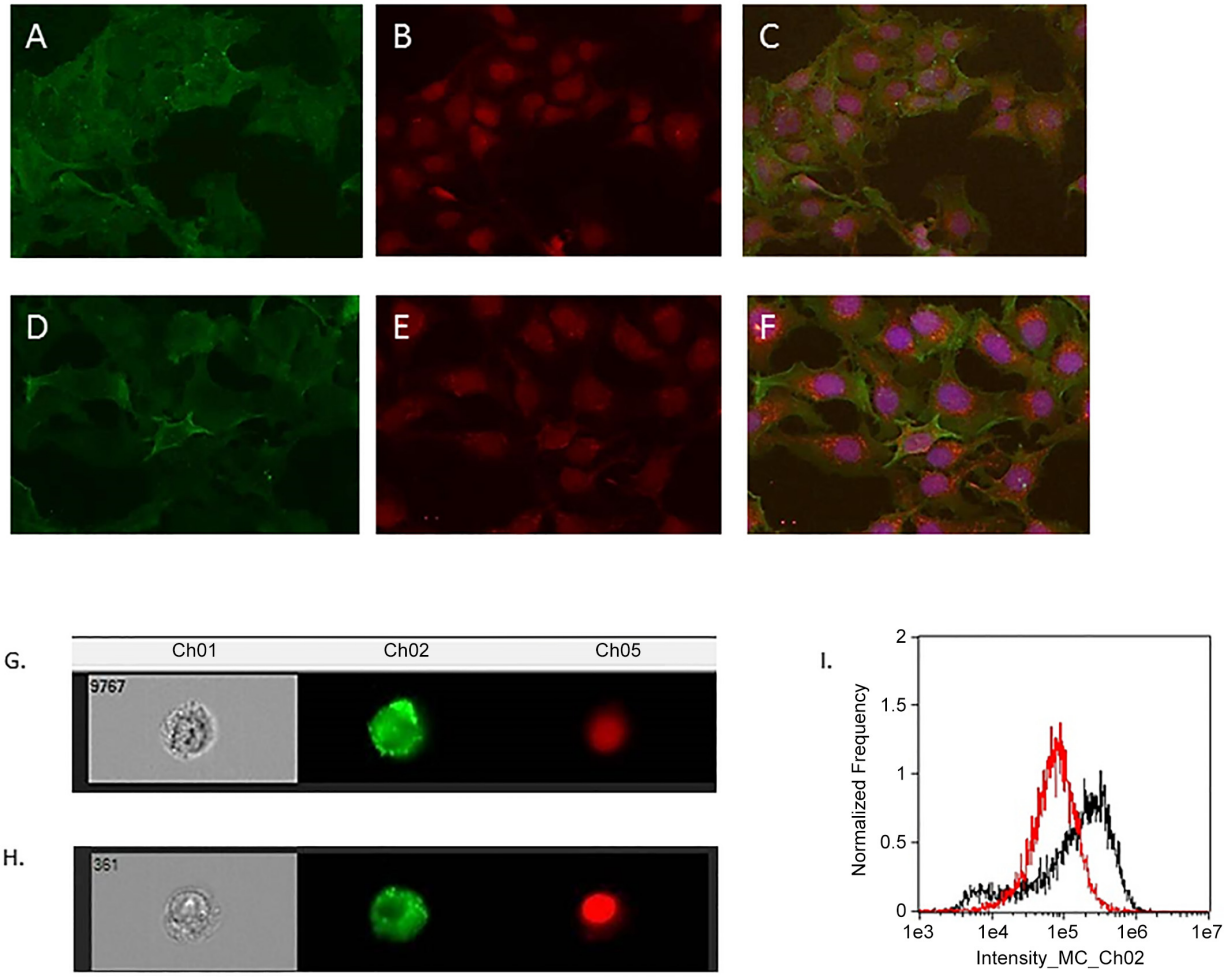
Immunofluorescent analysis of ULTRs, immunostained for SP-A (A) and SP-D (D). Nuclear staining using phalloidin 488 for SP-A (B) and SP-D (E). Merged images for SP-A (C) and SP-D (F). x 40 magnification. (G) and (H): Representative cells immunostained for SP-A and SP-D respectively. Merged image (I) showing the comparison of the fluorescence intensity of the cells between SP-A (red) and SP-D (black). SP-D immunostained cells appeared to have higher intensity compared to SP-A immunostained cells.

Using immunofluorescent analysis we demonstrate that ULTR cells express SP-A (Fig 6A–6C) and SP-D (Fig 6D–6F) aberrantly, with a predominant cytoplasmic localisation. We expanded on these analyses using high-power imaging technology. We have measured over 10,000 using ImageStream and it is evident that the expression is primarily on the cytoplasm for both proteins (Fig 6G and 6H). There was a higher fluorescence intensity SP-D immunostained cells appeared to to SP-A immunostained ULTR cells (Fig 6I).

**Fig 7:**
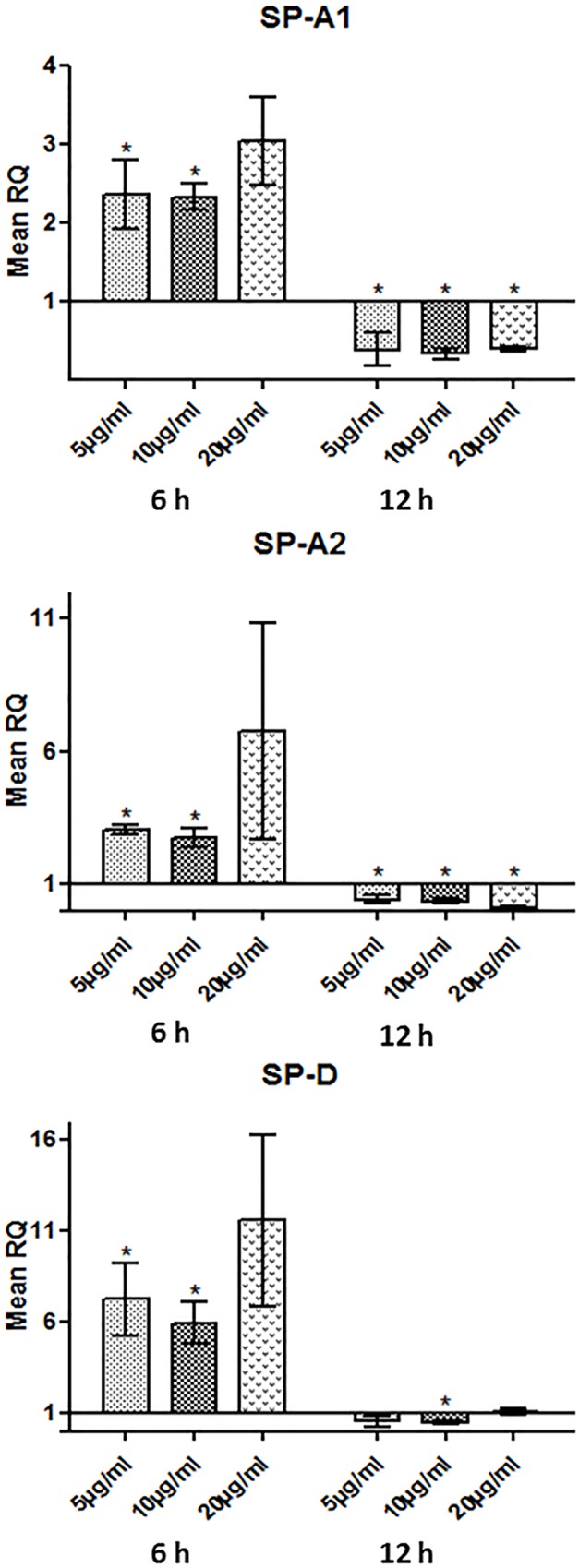
Relative quantification comparisons of SP-A1 (A), SP-A2 (B), and SP-D (C) in ULTR treated with 5, 10 and 20 μg/ml of rhSPD after 6 and 12h (*p<0.05, **p<0.01, ***p<0.001). rhSP-D treatments resulted in an increase of SP-A1, SP-A2 and SP-D mRNA expression at 6 h at 5 and 10 μg/ml. The effect was inverted at 12 h, with rhSP-D leading to a decrease in the expression of the above genes (n = 3).

Under the same treatment conditions of ULTR cells, a biphasic response was observed. rhSP-D induced mRNA expression of human SP-A1 (Fig 7A), SP-A2 (Fig 7B) and SP-D (Fig 7C) at a concentration of 5, 10 and 20 μg/ml at 6 h, followed by a moderate, but significant, decrease at 12 h post-treatment. rhSP-A led to a decrease in the expression of the SP-A transcripts after 6 h but did not have an effect on SP-D mRNA expression (rhSP-A data not shown). Treatments with either protein did not seem to affect the expression levels of COX2 (data not shown). Recent studies from our laboratory have also shown that the human myometrium differentially expresses mTOR signaling components. mTOR and DEPTOR mRNA levels did not seem to alter following treatment with either rhSP-A or rhSP-D (data not shown).

### Induction of growth factors and cytokines by rhSP-A and rhSP-D

**Fig 8:**
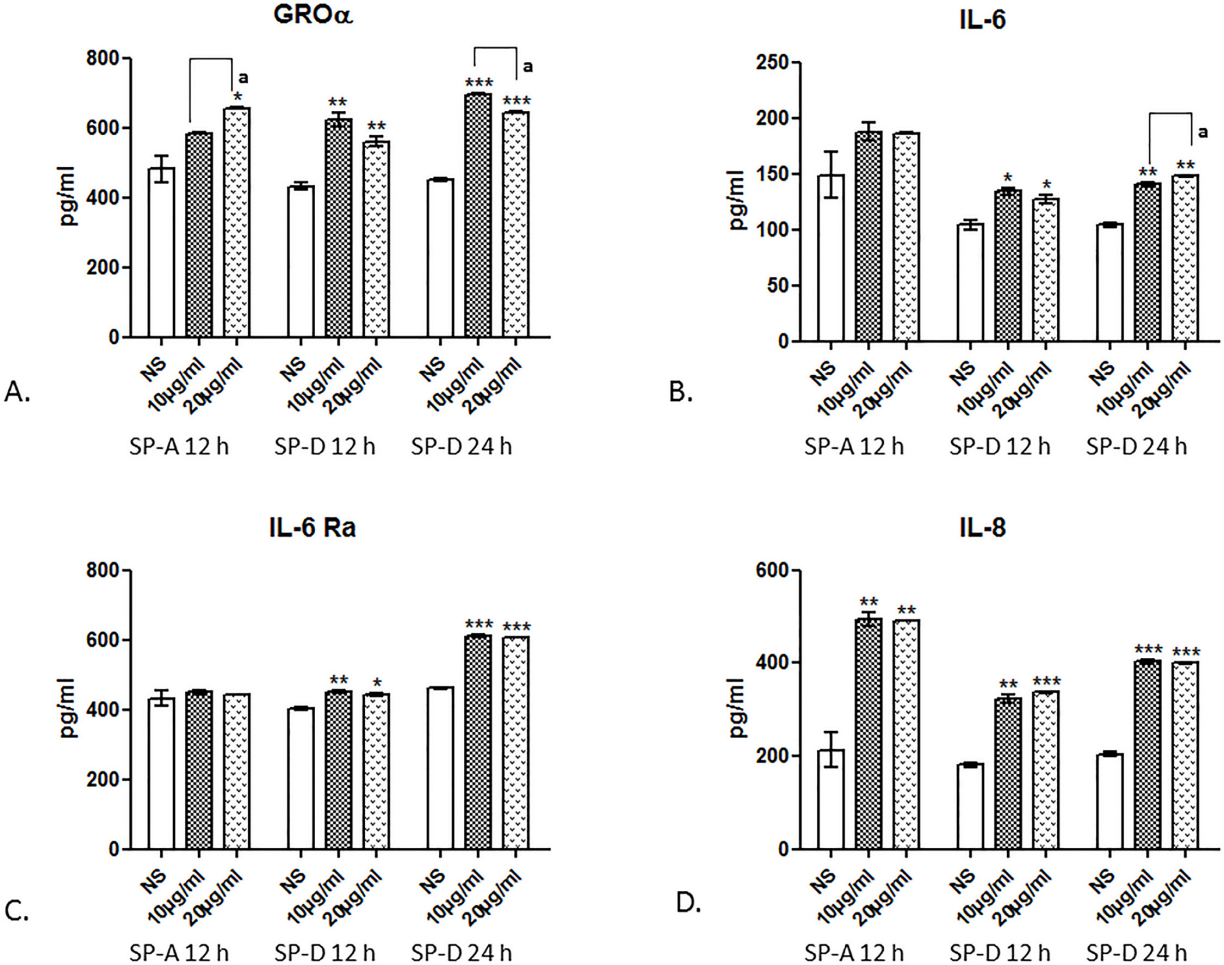
Multiplex cytokine array analysis of the supernatants of ULTR cells treated with rhSPA or rhSPD at different concentration time points (n = 2). Secretion levels for GROα (A), IL-6 (B), IL-6 Ra (C) and IL-8 (D), were measured by a MagPix Milliplex kit (EMD Millipore) rhSP-A treatments (10 and 20 μg/ml) caused an increase in the expression of GROα and IL-8 compared to the untreated cells, but did not have a profound effect on IL-6 and IL-6Ra. rhSP-D treated cells (10 and 20 μg/ml) appeared to express higher levels of GROα, IL-6 and IL-8 at 12 and 24 h compared to the untreated cells. ULTR cells treated with rhSP-D (10 and 20 μg/ml) expressed higher levels of IL-6 Ra after 24h. (*p<0.05, **p<0.01, ***p<0.001). Letter denotes values significantly different from each other as indicated by the horizontal and vertical lines (p<0.05).

To investigate the effects of surfactant protein treatments on the cytokine expression in ULTR cells, a multiplex cytokine array was used. FGF and VEGF play a key role in various cell developmental and pathophysiological changes *in vivo* and *in vitro* [26]. rhSP-A did not seem to affect the expression levels of either FGF or VEGF, whereas rhSP-D induced secretion of both cytokines/growth factors. Surprisingly, treatments with rhSP-A and rhSP-D appeared to have little or no effect on the secretion of IFN-γ, TNF-α and IL-1β which are important pro-inflammatory cytokines during the first trimester of pregnancy [27]. Both treatments led to an increase of ENA-78, also called C-X-C motif cytokine 5, which is concurrently expressed with IL-8 and promotes angiogenesis and tissue remodelling [28]. Treatments with rhSP-A led to an increase in the expression of GROα (a chemokine elevated in preterm labour) after 12 h at 10 and 20 μg/ml (1.2 and 1.4-fold respectively). rhSP-D treatments (10 and 20 μg/ml) led to a 1.4 and a 1.3-fold change respectively after 12 h and a 1.5 and 1.4-fold change after 24 h (Fig 8A). Our treatments had a similar effect on the pro-inflammatory cytokines IL-6 and IL-8. rhSP-D treatments at both concentrations appeared to result in an increase of IL-6 expression (1.3-fold change at 10 μg/ml, 1.2-fold change at 20 μg/ml after 12 h and a 1.3-fold change at 10 μg/ml, 1.4-fold change at 20 μg/ml after 24 h). IL-8 expression was increased by 2.3-fold at both concentrations at 12 h in ULTR cells treated with rhSP-A, whereas ULTR treated cells with rhSP-D increased expression of IL-8 by 1.8 and 1.9-fold at 12 h and by 2-fold at 24 h (Fig 8B–8D). IL-6 and IL-8 have been shown to promote immune infiltration, angiogenesis and regulate various aspects of the immune response [29]. In addition, levels of IL-6 Ra, which is a soluble receptor that binds to IL-6 in solution and augments its activity, were also increased after treatment with rhSP-D after 12 h (1.1-fold increase) and 24 h (1.3-fold increase) compared to the control (Fig 8C).

## Discussion

It has been proposed that surfactant proteins SP-A and SP-D secreted into the amniotic fluid can play a key role in parturition. In this study, we elucidated the effects of SP-A and SP-D using a human myometrial cell line as an experimental model. For this study we have decided to use the myometrial ULTR cell line, since due to ethical considerations we could not obtain primary myometrial cell cultures. ULTR is is a smooth muscle cell line that expresses all the key components of the myometrium and maintains the features of primary human myometrial cells [30]. Moreover, previous studies have used this *in vitro* model to study myometrial function and cytokine release as they have similar response as primary myometrial cells [30, 31]. In the future, it will be of interest to repeat these experiments in primary human myometrial cells.

We demonstrate that the human myometrium is a source of SP-A and SP-D, and that they can affect cell motility, and trigger contractility by inducing contraction-associated protein (CAP) genes such as OXTR and CX43 and augmenting the secretion of pro-inflammatory cytokines, such as IL-6 and IL-8.

The myometrium undergoes substantial changes in phenotype during the gestational period. It begins with a proliferative phase, followed by cellular hypertrophy and remodelling of matrix, and finally a phase where myometrial cells acquire a contractile phenotype and drive events that lead to labour and parturition [2, 24]. Here we demonstrate that both rhSP-A and rhSP-D were able to increase distance and velocity and cell displacement of myometrial cells. In the wound healing assay where ULTR cells treated with rhSP-A closed the gap at 17 h whereas ULTR cells treated with rhSP-D did so after 25 h. These data indicate that both SP-A and SP-D can play an important role in the reconditioning of the uterus particularly during the intermediate synthetic phase. Our data are further corroborated by initial studies where SP-A was able to induce F-actin stress fibres in primary myometrial cells [32]. Collectively, these findings suggest that SP-A in particular can play a key role in myometrial cytoskeleton reorganisation. A number of studies have shown that surfactant proteins are present in intra-uterine tissues including myometrium [32, 33], amniotic fluid and fetal membranes [16], decidua and placenta [15]. It is possible therefore that these peptides can exert their effects in an autocrine or paracrine manner. For example, corticotropin releasing hormone is produced in a similar fashion from a number of intra-uterine organs–including the myometrium, decidua and placenta- and exerts its effects in endocrine, paracrine and autocrine manner [34, 35].

The mechanistic target of Rapamycin (mTOR) appears to be involved in the reconditioning of the myometrium during early stages of gestation in rodents [36]. Moreover, we have shown that the human myometrium differentially expresses mTOR signaling components that are regulated by steroids [24]. When myometrial cells were treated with rhSP-A or rhSP-D, no apparent changes on mTOR or DEPTOR gene expression were noted. Further studies are needed to investigate whether SP-A and SP-D can affect mTORC1 activity by changing the phosphorylation status of key proteins like mTOR or S6K.

We also assessed the effects of both surfactant proteins on contraction-associated protein (CAP) genes. rhSP-A and rhSP-D induced the expression of OXTR and CX43 at 4 and 6 h. The induction of CX43 was greater than that of OXTR by at least 2-fold. Interestingly, female mice doubly deficient in SP-A and SP-D (SP-A/D^-/-^) have been shown to exhibit significantly lower levels of OXTR and CX43 at 18.5 dpc compared with wild-type (WT) in their myometrium [37]. We expanded further on these observations by measuring an actual contractile response using myometrial cells grown on 3D collagen matrices. At 3 h post-treatment, there was a dramatic induction in contractility, an effect sustained up to 24 h. Both surfactant proteins elicited a similar degree of response. These novel data using a 3D human *in vitro* model corroborate previous *in vivo* studies in mice where intraamniotic injection of SP-A at 15.5 dpc caused preterm delivery of foetuses within 6 to 24 h. By contrast, injection of an SP-A antibody into the amniotic sac of mice delayed labour by more than 24 h [18]. In a more recent study using SP-A/D^-/-^ mice, females delivered at term (∼19.5 dpc) in their first pregnancy, but in their second pregnancies, parturition was delayed by approximately 12 h [37].

Both term and preterm labour are associated with increased levels of pro-inflammatory cytokines such as IL-1β, IL-6, IL-8, and TNF-α in maternal sera and reproductive tissues [38]. Preterm birth, in particular, can affect up to 18% of pregnancies and is a leading cause of infant morbidity and mortality [39]. Here we have used conditioned media from myometrial cells treated with SP-A and SP-D and assayed a wide repertoire of cytokines using a multiplex cytokine array. Cytokines that were raised included GRO alpha (GROα), ENA-78, IL-6 Ra, IL-6 and IL-8. This is of particular significance since GROα has a chemotactic activity for neutrophils. Królak-Olejnik *et al*., have shown that GROα is lower in full-term infants than in preterm ones [40]. More importantly, the key pro-inflammatory cytokine, IL-8, was also raised by rhSP-A and rhSP-D. In rhSP-D treated cells, there was also a noted increase in IL-6 and the soluble IL-6 Rα that can potentially bind to IL-6 in solution and augment the activity of IL-6. In the SP-A/D^-/-^ mice, IL-6 levels were reduced in the myometrium but not in the amniotic fluid when compared to wild type female mice [37]. In a more recent study, it was shown that co-culture of monocytes and myocytes induces an increased pro-inflammatory response and myometrial contraction, involving IL-6 and IL-8h [41]. Future studies should make use of full length recombinant SP-A and SP-D, together with full length native C1q to provide a further insight into their role at myometrial level.

In summary, our novel findings provide conclusive evidence for a key role of both SP-A and SP-D in reconditioning the human myometrium and inducing CAP genes and inflammatory cytokines. This is intended to cause a shift of the uterus from a quiescent state to a contractile one leading to parturition.

## Supporting Information

S1 File Expression and characterisation of recombinant fragment of human SP-A (rhSP-A). SDS-PAGE analysis of rhSP-A at different stages of purification **(Fig A).** 15% (w/v) SDS-PAGE analysis for BS3 cross-linking of recombinant SP-A **(Fig B).** Western blot analysis of rhSP-A **(Fig C).**
(DOCX)
