# Supplementary material for: Surfactant Proteins SP-A and SP-D Modulate Uterine Contractile Events in ULTR Myometrial Cell Line
Source: PLoS One. 2015 Dec 7;10(12):e0143379. doi: 10.1371/journal.pone.0143379 (PMC4671565; doi:10.1371/journal.pone.0143379)
Supplement: S1 File — SDS-PAGE analysis of rhSP-A at different stages of purification (Fig A). 15% (w/v) SDS-PAGE analysis for BS3 cross-linking of recombinant SP-A (Fig B). Western blot analysis of rhSP-A (Fig C). (DOCX) [file pone.0143379.s001.docx]

**S1 File. Expression and characterisation of recombinant fragment of human SP-A (rhSP-A)**

**Cloning, expression and purification of a recombinant fragment of human SP-A (rhSP-A) containing homotrimeric neck and carbohydrate recognition domain:** Using full length SP-A1 gene template sub-cloned in pcDNA, the two terminal primers CACCATGCATCTAGATGAGGAGCTCCAAGC and GGGCTCGAGTCAGAACTCACAGATGGTCAGTCG were used to amplify the fragment containing neck and CRD region. The 444 bp was cloned in pET101/D-TOPO via recreated XhoI site. The recombinant proteins containing trimeric lectin domains were expressed in Escherichia coli BL21 (λDE3) pLysS (Life Technologies, UK). The bacterial cells were grown in Luria-Bertani medium with 100 µg/ml ampicillin and 34 µg/ml of chloramphenicol, shaking at 37°C until an A600 of 0.6-0.8 is reached, following an induction with 0.4 mM isopropyl β-D-thiogalactoside (IPTG), left for 3 h shaking at 37°C. The cells were centrifuged 4500rpm, 4°C, for 10 min. The cell pellet was suspended in lysis buffer (50 mM Tris-HCL pH 7.5, 200 mM NaCl, 5 mM EDTA, 0.1% v/v Triton X-100, 0.1 mM PMSF, 50 μg lysozyme) for 1 hour, followed by sonication using a Soniprep 150 (MSE, London, UK) at 60 Hz for 30 seconds with an interval of 2 min (12 cycles), which was then centrifuged at 12000 rpm for 15 minutes. The pellet was solubilized in 50 ml buffer A (50 mM Tris-HCl pH 7.5, and 100 mM NaCl) with 10 mM 2-mercaptoethanol (Bio-Rad, Hertfordshire, UK) and 8 M urea for 1 hour at 4°C. The soluble fraction was dialysed against a gradient of buffer A containing 4 M urea, 2 M urea, and 1 M urea for 2 hours at each urea concentration, which was then dialysed against affinity buffer (50 mM Tris-HCl pH 7.5, 100 mM NaCl, 10 mM CaCl2) externally with 2 changes and then centrifuged (10,000 rpm, 10 minutes, 4°C). The supernatant was then passed through 5ml column of maltose-agarose column, which was washed 3 column volumes of affinity buffer. Bound rhSP-A was eluted with buffer A consisting of 10 mM EDTA. Samples collected at each stage of purification were analysed on SDS-PAGE (S1 Fig).


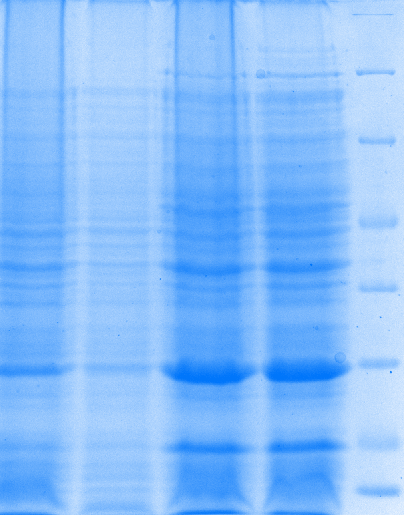

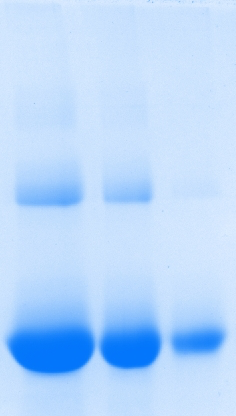


14.4

18.4

35.0

25.0

45.0

66.2

1 2 3 4 5 6 7 8


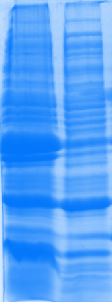


**Fig A. SDS-PAGE analysis of rhSP-A at different stages of purification.** A difference in samples for 3h before induction in lane 1, just before inducing in lane 2, and induced with IPTG lane 3 and lane 4 shows an overexpressed band at 18 kda for SP-A. Lane 5 – protein marker, lane 6 – pellet after lysis, lane 7 – supernatant after lysis, lane 8 – purified fraction of SP-A after maltose agarose column.

**Removal of LPS contamination from purified rhSP-A:** 5 ml of Polymyxin B agarose gel (Sigma, Poole, UK) in a 20 ml BioRad column was prepared to remove LPS of rhSP-A. The column was washed with 50 ml of 1% sodium deoxycholate and then washed with 50 ml of sterile dH2O. Peak fractions of rhSP-A or rhSP-D were applied to LPS removal columns for 2 h at 4°C. 1 ml fractions of the flow through were collected of the proteins. The levels of endotoxin were determined by QCL-1000 Limulus amebocyte lysate system (BioWhittaker, Walkersville, MD, USA). The concentration of protein fractions was measured at A280 using a NanoDrop. The endotoxins levels was examined by QCL-1000 LAL assay (BioWhittaker, Walkersville, MD, USA) and was found to be ~ 4 pg μg^-1^ of rhSP-A.

# Cross-inking assay: In order to establish if the rhSP-A were forming a trimeric structure, a chemical cross-linking method was used via Bissulfosuccinimidyl suberate BS^3^(in addition to gel filtration). 5μl of 0.01mM, 0.1mM and 1.0mM concentration of BS^3^ was incubated with 45μl of purified protein for 1 min, 2 min and 4 min at room temperature, and equal volume of treatment buffer was added at each time point. The samples for cross linking reaction were then left at 95°C for 10 min, and analyzed on 15% SDS PAGE (S2 Fig).


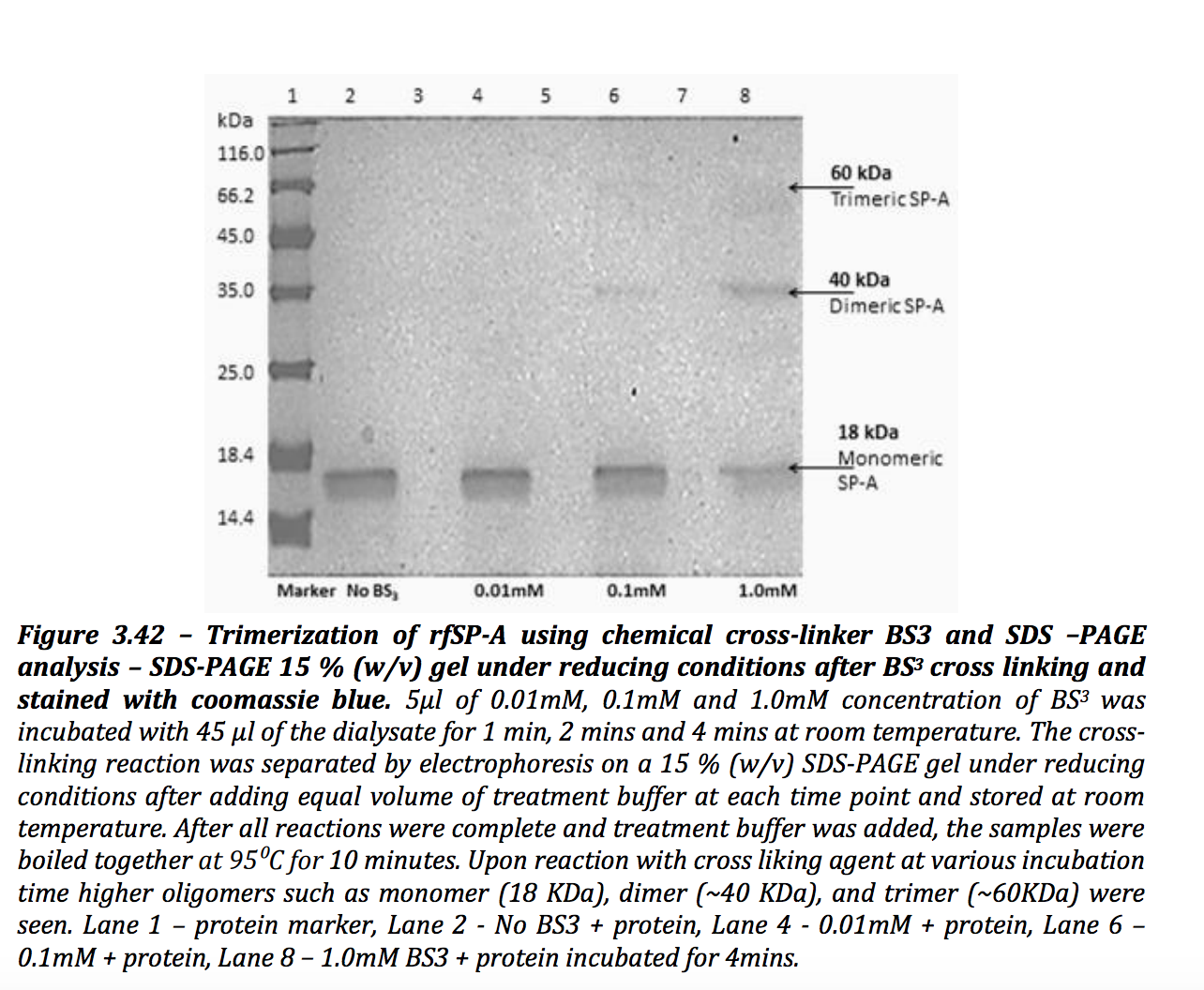


**Fig B. 15% (w/v) SDS-PAGE analysis for BS^3^ cross-linking of recombinant SP-A.** 5μl of 0.01mM, 0.1mM and 1.0mM concentration of BS^3^ was incubated with 45μl of dialysate for 1 min, 2 min and 4 min at room temperature, and equal volume of treatment buffer was added at each time point. The samples for cross linking reaction were then left at 95°C for 10 min, and analyzed on 15% SDS PAGE. The monomer at 18 kDa, dimer at 40 kDa, and trimer at ~60 kDa can be observed. Lane 1: protein marker; lane 2: no BS^3^ + protein; lane 4: 0.01mM BS^3^+ protein, lane 6: 0.1mM BS^3^ + protein, lane 8: 1.0 mM BS^3^ + protein.

**Western blotting to test immunoreactivity of rhSP-A:** 5 μg or rhSP-A was loaded in two separate lanes and SDS-PAGE was run until the dye front reached the end of the gel. The proteins were then transferred on to a nitrocellulose membrane by electrophoresis in 1 x transfer buffer (25mM Tris, 192mM Glycine, 20% v/v methanol, pH ~8.3) at 320 mA for 2 h. The membrane was incubated in blocking solution, 5% w/v semi-skimmed milk powder in PBS, pH 7.4 to reduce non-specific binding overnight at 4°C. The membrane was then washed in PBST three times for 10 min each. Rabbit anti-human SP-A polyclonal antibodies (raised against full length native SP-A purified from human lung lavage) (1:2500) in PBS was incubated with the membrane for 1 h at room temperature. The membrane was washed again with PBST as above. The secondary probe, Protein A-HRP (1:5000 dilution) (Thermo Scientific) was added to the blot and incubated for 1 h at room temperature. The secondary antibody was discarded and membrane was washed with PBS Tween 20, 0.02% thrice for 5 mins each. 3,3′-Diaminobenzidine (DAB) tablets were dissolved in 15ml dH_2_O and the membrane was incubated for 10 mins to visualise the bands (S3 Fig). BSA was used as a negative control that did not show up on western blot.


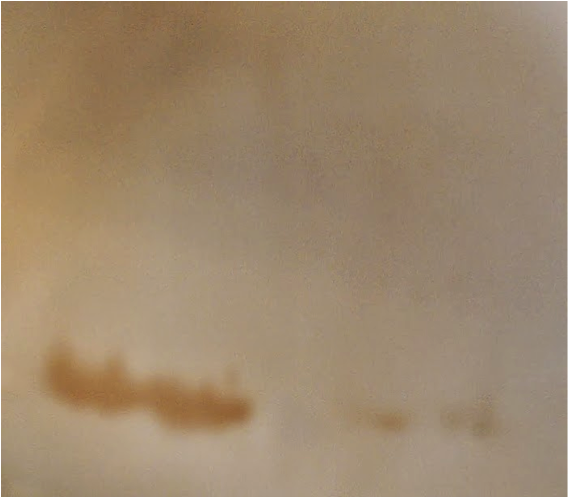


18 kDa

**Fig C.** **Western blot analysis of rhSP-A.** Purified rhSP-A (18 kDa) was verified by western blot by probing with anti-human SP-A polyclonal antibodies.
